# The weighing process in patients on hemodialysis: an opportunity to improve volume management

**DOI:** 10.1093/ckj/sfae275

**Published:** 2024-09-03

**Authors:** Janosch Niknam, Sebastian Mussnig, Christoph Matthias, Maximilian Waller, Nikolaus Keil, Simon Krenn, Joachim Beige, Daniel Schneditz, Manfred Hecking

**Affiliations:** Medical University of Vienna, Department of Medicine III, Division for Nephrology and Dialysis, Vienna, Austria; Medical University of Vienna, Center for Public Health, Department of Epidemiology, Vienna, Austria; Medical University of Vienna, Department of Medicine III, Division for Nephrology and Dialysis, Vienna, Austria; Medical University of Vienna, Center for Public Health, Department of Epidemiology, Vienna, Austria; Medical University of Vienna, Center for Public Health, Department of Epidemiology, Vienna, Austria; AIT Austrian Institute of Technology GmbH, Center for Health & Bioresources, Medical Signal Analysis, Vienna, Austria; Medical University of Vienna, Center for Public Health, Department of Epidemiology, Vienna, Austria; Klinik Favoriten, Department of Medicine I with Nephrology, Intensive Medicine, Psychosomatics and Diabetology, Vienna, Austria; Medical University of Vienna, Center for Public Health, Department of Epidemiology, Vienna, Austria; Klinik Favoriten, Department of Medicine I with Nephrology, Intensive Medicine, Psychosomatics and Diabetology, Vienna, Austria; Medical University of Vienna, Center for Public Health, Department of Epidemiology, Vienna, Austria; AIT Austrian Institute of Technology GmbH, Center for Health & Bioresources, Medical Signal Analysis, Vienna, Austria; Kuratorium for Dialysis and Transplantation (KfH), Germany; Medical University of Graz, Division of Physiology & Pathophysiology, Otto Loewi Research Center for Vascular Biology, Immunology and Inflammation, Graz, Austria; Medical University of Vienna, Department of Medicine III, Division for Nephrology and Dialysis, Vienna, Austria; Medical University of Vienna, Center for Public Health, Department of Epidemiology, Vienna, Austria; Kuratorium for Dialysis and Transplantation (KfH), Germany

**Keywords:** body mass, clothing mass, hemodialysis, ultrafiltration, volume management

## Abstract

**Background:**

Hemodialysis relies on accurate body mass (BM) assessment to determine ultrafiltration volumes, but we have not identified published practice patterns disclosing how to handle clothing mass. Here we investigated the potential impact of clothing mass on predialysis BM determination, hypothesizing that a standardized template for clothing mass estimation enhances accuracy, compared with conventional practice.

**Methods:**

Measurements included dressed and undressed BM predialysis. A pre-established template for average clothing mass was used to approximate undressed BM from clothed measurements. Differences from undressed BM were compared using Bland–Altman plots and tested for statistical significance using Wilcoxon signed rank tests.

**Results:**

After excluding erroneous results, data from 48 patients were analyzed. Thirty-six patients (75%) did not habitually estimate clothing mass, but used their dressed BM as the predialysis BM, while the other 12 patients (25%) reported deducting a self-estimated clothing mass from their clothed predialysis BM. The differences from undressed BM were 0.819 ± 0.462 kg and 0.342 ± 0.321 kg in these two groups, respectively, indicating that patients underestimated clothing mass. Using the template to deduct clothing mass from clothed predialysis BM, these differences could be reduced to 0.197 ± 0.220 kg and 0.133 ± 0.135 kg, respectively. The average differences using the patient-reported BM and the template-based BM made up 39.4% and 8.6% of the average subsequent ultrafiltration volume, respectively, suggesting that potential overestimation of the actual ultrafiltration volume could be reduced.

**Conclusion:**

A standardized template for clothing mass may be useful to derive representative predialysis BM, leading to more precise ultrafiltration calculation. Exact BM determination might improve volume management in hemodialysis.

KEY LEARNING POINTS **What was known:**

- Hemodialysis requires precise predialysis body mass (BM) assessments to calculate ultrafiltration volume.
- No standardized method accounts for clothing mass, posing risks to patient safety and treatment effectiveness.
- The impact of clothing mass on predialysis BM determination is underexplored, indicating a gap in optimizing dialysis practices.

**This study adds:**

- Utilizing a standardized template for estimating clothing mass significantly improves the accuracy of predialysis BM estimates, thereby refining the calculation of ultrafiltration volume.
- It highlights the common underestimation of clothing mass in current practices and introduces a practical, implementable solution for dialysis centers to standardize predialysis weighing procedures.

**Potential impact:**

- Integrating a standardized clothing mass template in hemodialysis could enhance fluid management accuracy and thereby minimize complications from incorrect ultrafiltration volumes.
- This method may offer a direct path to safer, more precise dialysis care, with implications for improving clinical outcomes and practice standards.

## INTRODUCTION

Hemodialysis (HD) is used to restore the normal state of intracellular and extracellular electrolyte and fluid balance in patients with kidney failure. Apart from clearance of metabolites and adjustment of acid–base balance, an equally important function of HD is to remove excess fluid volume via ultrafiltration [1]. The amount of fluid volume that must be removed during dialysis is usually determined by comparing the predialysis BM with the target BM at which the patient is considered to be euvolemic. Interdialytic weight gain (IDWG), the difference between predialysis BM and the postdialysis BM of the preceding dialysis session, is primarily explained by net fluid and food intake, and is commonly assumed to represent volume that should be removed during the subsequent treatment. IDWG and the current difference from the target BM are determined prior to each dialysis session and are used to calculate the ultrafiltration volume for the upcoming dialysis session [2–4]. The ultrafiltration rate is then derived from the prescribed ultrafiltration volume divided by the dialysis duration. Ideally, this rate should not exceed 13 mL/h/kg, since high ultrafiltration rates are associated with risks of intradialytic complications, all-cause mortality and cardiovascular mortality [5–9].

As ultrafiltration volumes are primarily dictated by BM dynamics, accurate assessment of BM is of major importance. Inaccuracies could increase risks of intradialytic hypovolemia or chronic volume overload. Our previous experience from multiple HD units in Austria and Germany suggest that there is no standardized practice pattern to derive accurate predialysis BM for all dialysis centers, and indeed not even for a single dialysis center. Some patients may weigh-in fully clothed and use this measurement, while others may deduct an arbitrary mass for their garments. To our best knowledge, there are no published data on the weighing process of dialysis patients. In daily routine of hemodialysis units, the weighing process including the patient’s workarounds to reach personally desired mass estimation sometimes do not correspond to the necessary accuracy that should be obtained for such an important process. We hypothesized that deducing the session-specific ultrafiltration volume from BM dynamics might be distorted depending on the patients’ weighing procedure, even when accounting for a “constant” estimated clothing mass. Although this error could be avoided by always determining the actual bare BM before dialysis, such an approach is not feasible, especially for elderly and multimorbid patients.

In the present study, we therefore investigated whether the accuracy of estimated unclothed patient BM could be improved by using a clothing template compared with the current non-standardized weighing procedure.

## MATERIALS AND METHODS

### Study population, study design and ethics approval

The present study is an exploratory, hypothesis-generating, retrospective data analysis. From March to April 2023, patients undergoing maintenance HD treatment at the Chronic Hemodialysis Unit of the Vienna General Hospital (Vienna, Austria) underwent predialysis weighing procedures in addition to standard practice as part of a one-time clinical assessment. Patients were selected for these measurements via purposive sampling, especially taking into consideration their apparent mobility. Approval for this retrospective study was obtained from the Ethics Committee of the Medical University of Vienna (ethics committee number 1434/2023).

### Weighing procedures

**Figure 1:**
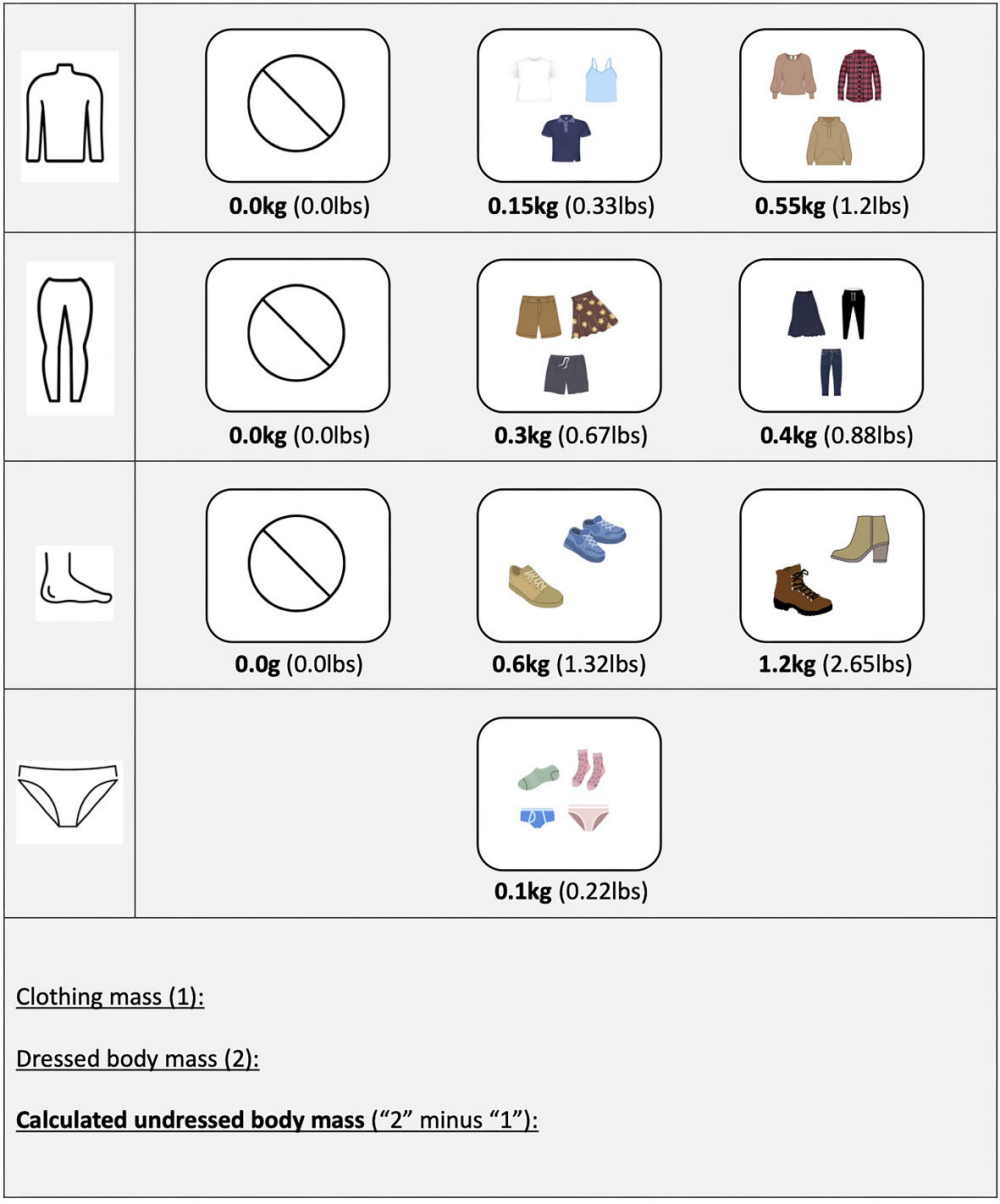
Template for estimating patient garment mass and calculating undressed BM. This visual template assists in estimating a patient's clothing mass. Icons represent typical garments with their average mass in kilograms and pounds. To estimate a patient's BM without clothes, the mass of the selected items are summed up (1) and subtracted from the dressed BM (2), resulting in the calculated undressed BM (“2” minus “1”).

Patients were weighed predialysis prior and in addition to their usual weighing process using the seca 954 chair scale (seca, Hamburg, Germany), which measures with a 0.045 kg graduation. First, measurements of dressed BM and mass of clothing items which were habitually removed by the patients were recorded. Patients were asked whether and how much they usually deduct for the mass of their clothes. Then, patients were asked to undress completely (except underwear), and this mass was recorded. A list of average garment masses was prepared before these assessments by weighing a wide range of clothing items which resulted in the creation of a template (Fig. 1). From these measurements, four different predialysis body masses (BM-dressed, BM-undressed, BM-reported and BM-template) were selected for further analysis, which are defined in Table 1. It should be noted that our measurements were conducted independently of the conventional weighing process and therefore were not used to determine the ultrafiltration volume of the respective session.

**Table 1:** Abbreviations of predialysis BM types with definitions and their respective determination method.

Abbrevation	Definition	Process
BM-dressed	BM with indoor clothes	Patients were weighed in the manner they were used to, wearing in-door clothing
BM-undressed	BM without clothes (underwear remained)	Patients were asked to remove all of their clothing, except for their underwear; 0.1 kg was deducted from the measured undressed BM to account for underwear
BM-reported	BM dressed with or without patient estimate of total garment mass. Input variable for predialysis BM to calculate IDWG and to determine ultrafiltration volume	After measuring BM-dressed, patients were asked if they normally deduct an estimated value for the remaining clothing. A distinction was made between [BM-reported (with deduction)] for those who deducted a self-estimated value for the remained clothing mass and [BM-reported (without deduction)] for patients who did not deduct anything
BM-template	BM with clothes minus the sum of worn garment items’ mass	A template with predefined average garment mass (Fig. 1) was used to determine the mass of the clothing patients had been weighed with. From each row, the correct box had to be selected (selection was based on the articles shown in the boxes) and all values were summed up to define the clothing mass. Then the clothing mass was subtracted from the dressed BM to obtain BM-template

### Data acquisition

Body masses from the additional weighing procedure were manually recorded from the chair scale. Pre- and postdialysis BM (recorded during the routine weighing procedure), target BM, ultrafiltration volume, ultrafiltration rate, dialysis session length and blood pressure values were automatically captured by Diamant Software Version 3.14, a dedicated data acquisition system (Diamant Software by Diasoft B.V., Leusden, The Netherlands). All data were merged to one .xlsx file using Microsoft Excel Version 16.80 (Microsoft, Redmond, WA, USA).

### Statistical methods

Metric variables were described by arithmetic means, standard deviations, medians and interquartile ranges, and were depicted in bar charts, boxplots and jitter plots. Categorical variables were reported as frequencies and percentages. Differences between predialysis body masses were visualized via Bland–Altman plots and were tested for statistical significance using the Wilcoxon signed rank test for paired data. *P*-values below .05 were considered to reject the null-hypothesis. *Post hoc* power analysis for analyses of differences between predialysis body masses was conducted because an *a priori* sample size calculation was lacking. Data analysis was conducted using Microsoft Excel, Jeffreys's Amazing Statistics Program—JASP Version 0.18.1 (University of Amsterdam, Amsterdam, The Netherlands) and R programming language Version 4.3.0 (2023-04-21) (The R Foundation for Statistical Computing, Vienna, Austria) using RStudio for macOS version 2023.09.0 + 463 (Posit Software, Boston, MA, USA).

### Online survey on weighing procedure

From 28 May 2024 onwards, we provided an online survey consisting of six questions about the predialysis weighing process to dialysis patients of the Kuratorium for Dialysis and Transplantation (KfH). Patients were prompted with an invitation to this survey upon connection to the local wireless network.

## RESULTS

### Patient characteristics and excluded data

**Figure 2:**
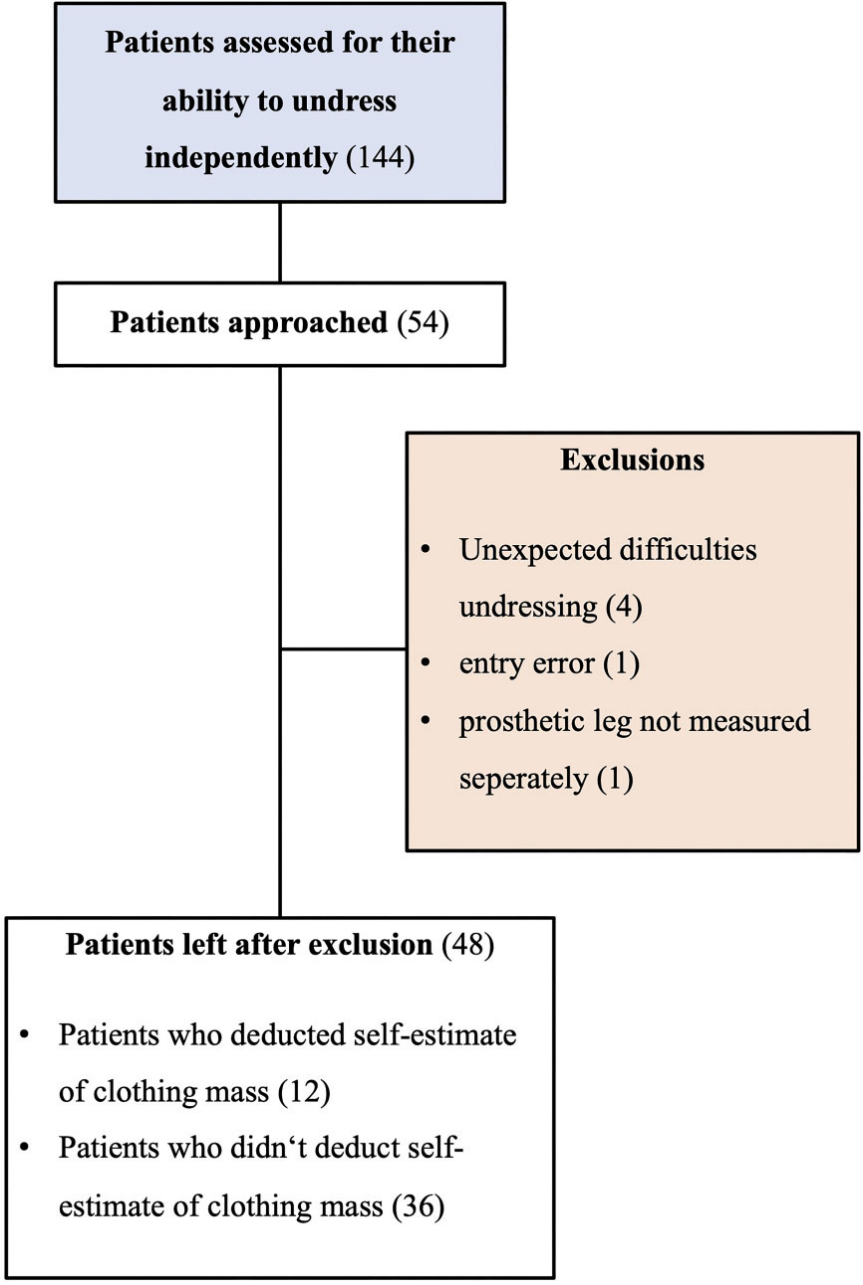
Patient flowchart.

**Figure 3:**
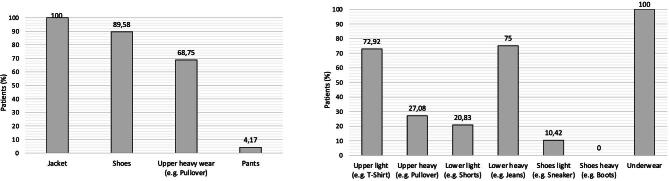
Percentages of patients who removed specific clothing items. Bar graphs with percentages of (**a**) clothing items removed before weighing and (**b**) clothing worn during weighing and documented using the template (% of patients). The reference group for each percentage is the entire patient population.

Between March and April of 2023, 144 patients underwent maintenance HD treatment at the study site. Of 54 dialysis patients who were approached, 48 were included for further analysis (Fig. 2), 21 of whom (43.8%) were women. Patient characteristics and dialysis data are shown in Table 2. The mean age ± standard deviation was 55.8 ± 15.8 years. Clothing items removed before weighing and clothing worn during the weighing process were recorded as part of the measurements and are shown in Fig. 3.

**Table 2:** Patient characteristics.

Variable	Value
Number of patients, *n*	48
Female sex, *n* (%)	21 (43.8)
Age, years	55.8 (15.8)
Biopsy-proven diabetic KD^a^ (1), *n* (%)	2 (4.2)
Glomerular KD^a^ (1), *n* (%)	14 (29.2)
Vascular KD^a^ (1), *n* (%)	3 (6.3)
Other cause for ESKD^a^ (1), *n* (%)	18 (37.5)
Unknown cause of ESKD^a^ (1), *n* (%)	10 (20.8)
Type 1 diabetes mellitus ^a^ (1), *n* (%)	2 (4.2)
Type 2 diabetes mellitus^a^ (1), *n* (%)	10 (20.8)
Congestive heart failure^a^ (1), *n* (%)	18 (37.5)
Arterial hypertension^a^ (1), *n* (%)	35 (72.9)
Coronary artery disease^a^ (1), *n* (%)	18 (37.5)
Cerebrovascular disease^a^ (1), *n* (%)	5 (10.4)
Peripheral arterial disease^a^ (1), *n* (%)	6 (12.5)
DVT, CVI^a^ (1), *n* (%)	3 (6.3)
Aortic aneurysm^a^ (1), *n* (%)	4 (8.3)
History of cancer^a^ (1), *n* (%)	9 (18.8)
Lung disease^a^ (1), *n* (%)	8 (16.7)
Neurologic disorder^a^ (1), *n* (%)	5 (10.4)
Psychologic disorder^a^ (1), *n* (%)	1 (2.1)
Predialysis BM^a^ (2), kg	73.4 (18.0)
Postdialysis BM^a^ (6), kg	69.8 (17.6)
Target BM^a^ (2), kg	70.5 (17.5)
UF volume^a^ (4), L	2.6 (1.1)
UF rate^a^ (4), mL/h	701.1 (315.9)
DSL^a^ (4), min	223 (29.9)
sBP (predialysis)^a^ (4), mmHg	145.1 (25.8)
dBP (predialysis)^a^ (4), mmHg	83.4 (23.0)
sBP (postdialysis)^a^ (4), mmHg	139.2 (25.8)
dBP (postdialysis)^a^ (4), mmHg	81.8 (17.2)
sBP (lowest intradialytic)^a^ (4), mmHg	130.1 (23.3)

Metric variables are listed as arithmetic mean and (standard deviation). Categorical variables are reported in absolute numbers and percentage of their respective group.

^a^(Number) of data are missing for this parameter.

KD: kidney disease; ESKD: end-stage kidney disease; DVT: deep vein thrombosis; CVI: chronic venous insufficiency; DSL: dialysis session length; dBP: diastolic blood pressure; sBP: systolic blood pressure; UF: ultrafiltration.

### Differences between predialysis BMs

**Figure 4:**
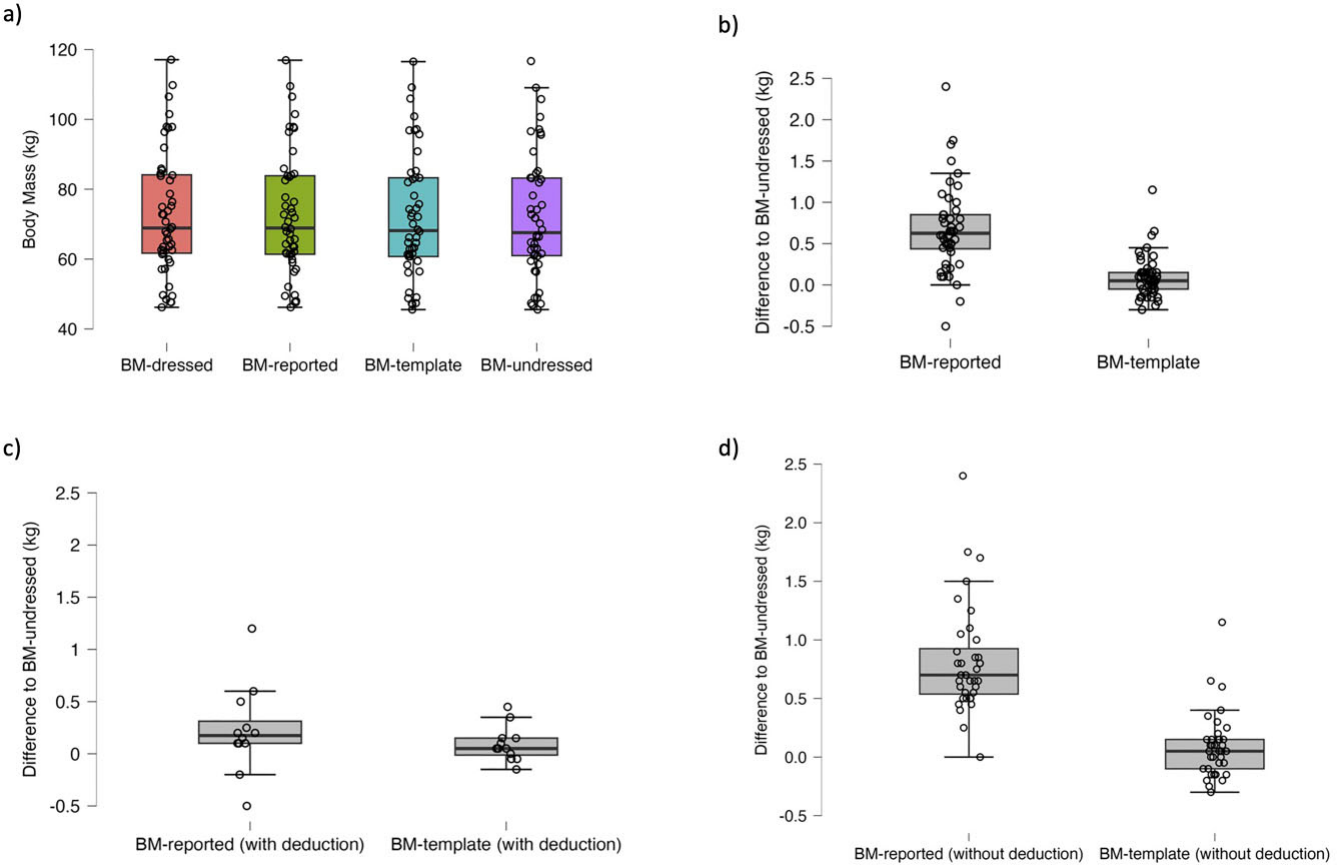
Boxplots and jitter plots of different predialysis BM measures. Boxplots overlayed with jittered individual datapoints for (**a**) predialysis body masses in kg; (**b**) comparison of BM-reported minus BM-undressed vs BM-template minus BM-undressed for all patients in kg; (**c**) comparison of BM-reported minus BM-undressed vs BM-template minus BM-undressed for patients who calculate their predialysis BM (BM-reported) by deducting a self-estimated value for clothing mass in kg; and (**d**) comparison of BM-reported minus BM-undressed vs BM-template minus BM-undressed for patients who did not deduct a self-estimated value for clothing mass in kg.

**Figure 5:**
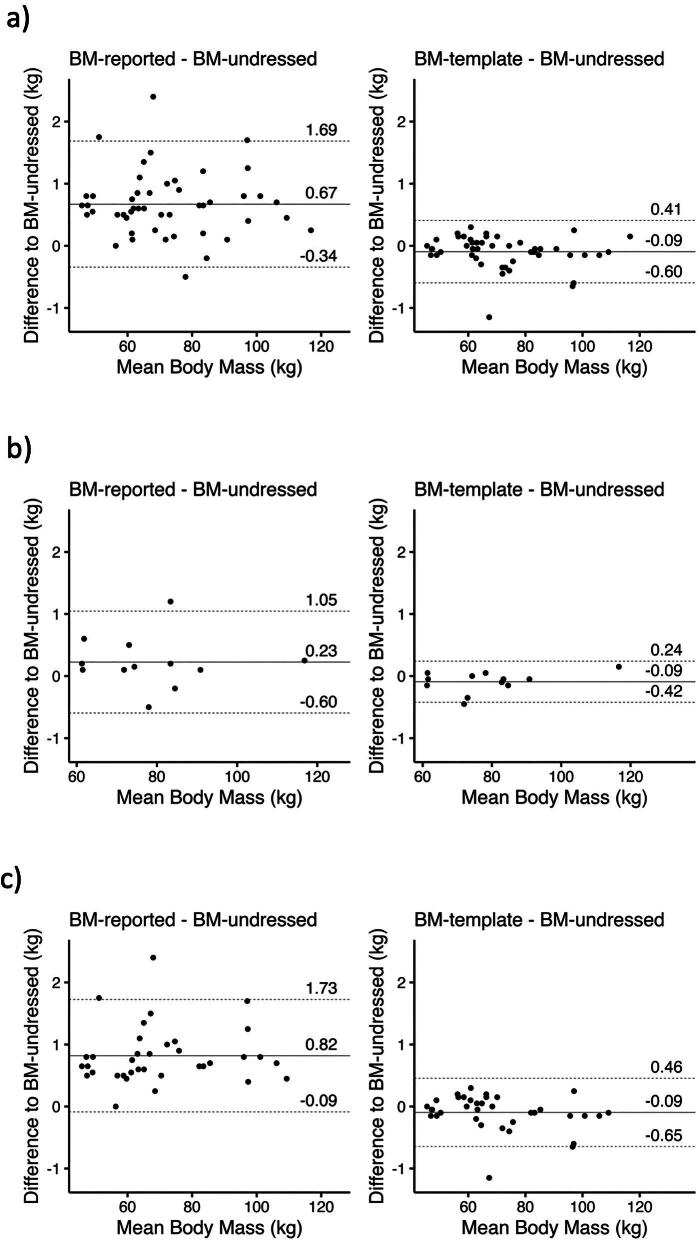
Bland–Altman plots of different predialysis body masses. Bland–Altman plots for BM-reported minus BM-undressed in kg (left) and BM-template minus BM-undressed in kg (right) of (**a**) all patients (*n* = 48), (**b**) patients who deduct a self-estimate of clothing mass (*n* = 12) and (**c**) patients who do not deduct a self-estimate of clothing mass (*n* = 36).

Differences between BM-dressed, BM-reported (both with and without deducted estimated clothing mass) and BM-template to BM-undressed are listed in Table 3. The average difference between BM-reported (the conventional method for determining predialysis BM) and BM-undressed was 0.70 ± 0.48 kg (*P* < .001), while the average difference between BM-template (the estimated undressed predialysis BM using the clothing template shown in Fig. 1) and BM-undressed was 0.18 ± 0.20 kg (*P* < .001) (Table 3, Fig. 4b). These differences were similar over the entire spectrum of recorded measurements (Fig. 5a) and amounted to an average of 39.4 ± 53.2% [median 24.7% (11.5, 39.6)] of prescribed ultrafiltration volumes using BM-reported, and 8.6 ± 10.3% [median 4.6 (2.5, 8.6)] of prescribed ultrafiltration volumes using BM-template, on an intra-patient level.

**Table 3:** Results of predialysis BMs and mass differences versus BM-undressed.

Variable	*n*	Mean	SD	Minimum	25%	Median	75%	Maximum
BM-dressed	48	72.89	17.79	46.20	61.70	68.90	84.13	117.10
BM-undressed	48	72.00	17.80	45.55	61.00	67.58	83.19	116.70
BM-reported	48	72.68	17.77	46.20	61.40	68.90	83.85	116.95
BM-reported (with deduction)	12	78.51	15.54	61.40	69.43	76.08	84.10	116.95
BM-reported (without deduction)	36	70.73	18.24	46.20	58.53	66.48	82.86	109.50
BM-template	48	72.10	17.84	45.55	60.75	68.15	83.28	116.55
BM-reported – BM-undressed	48	0.70	0.48	0.00	0.45	0.63	0.85	2.40
BM-template – BM-undressed	48	0.18	0.20	0.00	0.05	0.15	0.20	1.15
BM-reported (with deduction) – BM-undressed	12	0.34	0.32	0.10	0.14	0.20	0.50	1.20
BM-reported (without deduction) – BM-undressed	36	0.82	0.46	0.00	0.54	0.70	0.93	2.40
BM-template (with deduction) – BM-undressed	12	0.13	0.14	0.00	0.05	0.08	0.15	0.45
BM-template (without deduction) – BM-undressed	36	0.20	0.22	0.00	0.09	0.15	0.21	1.15

Values are given in kilograms. Mass difference between two different predialysis mass types are described as, for example, BM-reported minus (–) BM-undressed.

25%: 25th percentile; 75%: 75th percentile; SD: standard deviation.

### Impact on ultrafiltration rates

The impact of inaccurate weight assessment on prescribed ultrafiltration rate is illustrated by the following estimates of ultrafiltration under use of the differently assessed BMs: the use of BM-dressed and BM-undressed would have led to average ultrafiltration rates of 12.84 ± 8.17 and 8.82 ± 8.08 mL/min, respectively. The actual ultrafiltration rate for BM-reported was 11.84 ± 7.70 mL/min compared with 9.23 ± 8.23 mL/min if the BM-template had been used.

### Accuracy of patient-estimated clothing mass

Twenty-five percent of patients reported that they usually deduct a self-estimated value for their remaining clothing. In these patients, the mean difference between BM-reported and BM-undressed was smaller compared with those who did not deduct anything, but was still larger than the mean difference between BM-template and BM-undressed (mean ± standard deviation 0.34 ± 0.32 kg vs 0.13 ± 0.14 kg; Table 3, Figs 4c and 5b). In patients who did not deduct a self-estimate for clothing, these differences were 0.82 ± 0.46 kg vs 0.20 ± 0.22 kg (Figs 4d and 5c).

### Online survey

To further elaborate on the relevance of our findings, we conducted an online survey on the weighing process within the KfH and received 268 responses by 19 July 2024 (53 days).

Eighty-seven percent of survey patients attributed 4 or 5 points on a 1–5 rating scale (5 = very important) to the weighing process. Thirty-seven percent of patients reported unintentional errors to occur sometimes or more often, of whom 43% also replied to knowingly accept these occasionally, frequently or always. Further results are provided in Table 4.

**Table 4:** Results of online survey on weighing procedure.

Question	Answer in % participants (*n*)
How important do you consider the weighing process for the efficiency and tolerability of hemodialysis?	
1	6 (*n* = 17)
2	1 (*n* = 2)
3	6 (*n* = 17)
4	20 (*n* = 53)
5 (= very important)	67 (*n* = 179)
Please estimate the frequency of unintentional errors during weighing (due to more or less clothing or accessories, such as a watch or jewelry, than usual)	
Never	23 (*n* = 61)
Rarely	43 (*n* = 110)
Sometimes	21 (*n* = 56)
Often	13 (*n* = 34)
Always	3 (*n* = 7)
Have you ever knowingly accepted errors in weighing?	
Never	65 (*n* = 175)
Once	7 (*n* = 18)
Occasionally	24 (*n* = 64)
Frequently	3 (*n* = 8)
Always	1 (*n* = 3)
Do you regularly deduct the weight of your clothing from your total weight when you weigh yourself before dialysis or report your weight to the nurse or your doctor?	
No	65 (*n* = 173)
Yes	35 (*n* = 95)
Do you always dialyze in the same clothes, such as pajamas, so that the weight of the clothing does not need to be considered when weighing?	
No	39 (*n* = 104)
Yes	61 (*n* = 164)
How helpful would a guide with clothing symbols for estimating clothing weight be for you?	
1	29 (*n* = 77)
2	14 (*n* = 38)
3	16 (*n* = 44)
4	15 (*n* = 40)
5 (= very helpful)	26 (*n* = 69)

Percentages have been rounded to the nearest whole number.

## DISCUSSION

The accurate assessment of predialysis BM in HD patients is crucial for determining the appropriate ultrafiltration volume and rate during dialysis sessions. Our study investigated the potential impact of clothing on the accuracy of predialysis BM and its subsequent effect on the determination of ultrafiltration variables.

The conventional method of weighing patients with clothing on, as commonly practiced in many HD units, revealed a notable average deviation from bare BM of 0.70 kg . This discrepancy poses a potential risk, as inaccurate predialysis BM could lead to inappropriate ultrafiltration volumes and rates. In contrast, when using a template to estimate and account for clothing mass, the average deviation significantly decreased to 0.181 kg, suggesting that a standardized template for clothing mass could be a practical approach to improve the accuracy of predialysis BM determination.

Even for patients who reported deducting a self-estimated value for their remaining clothing, the deviation from undressed BM was still greater compared with using a template. This emphasizes the variability and uncertainty associated with individual estimations of clothing mass. Therefore, adopting a systematic approach, such as the use of a template, may mitigate the inaccuracy of patients’ deductions, provide more reliable predialysis BM measurements and help staff with an easy-to-apply routine in a somewhat disliked procedure.

The determination of IDWG is not as trivial as commonly assumed, since both measurements required for calculating this parameter may be associated with a clothing-related error, which are then carried over to the estimation of mass gain. Errors may cancel each other out, but they may also add up. Three types of uncertainty must be considered. The first error is related to the accuracy of the weighing scale, typically in the range of ±0.1 kg under ideal measuring conditions because of the resolution of the scale and the digital representation of measured mass. The second error is related to variable clothing worn on the occasion of the two measurements. It can be assumed that this error is comparable to the difference between the measured and the estimated mass of clothing in the range of 0.3 kg. A third error is related to the natural variability of euvolemic BM between measurements in the magnitude of 0.4–0.5 kg [10]. If all these variabilities are considered, the discrepancy between a true fluid gain and a measured BM gain may well be 1 L, in the worst case scenario. The implications of day-to-day BM variability in the prescription of ultrafiltration volume have been recognized recently and it therefore has been suggested to use a soft rather than a fixed and constant target BM for the prescription of ultrafiltration volume also for stable HD patients [11].

The impact of inaccurate predialysis BM determination is further illustrated by its potential effect on prescribed ultrafiltration volume and rate. Our analysis revealed that when deviations in predialysis BM were set in relation to the selected ultrafiltration volume, the conventional method showed a considerably higher percentage (39.4%) compared with the template-based approach (8.6%). Our analysis shows that typical inaccuracies in predialysis weight measurement can lead to higher ultrafiltration rates. These findings emphasize the clinical significance of accurate predialysis BM in optimizing ultrafiltration parameters and, subsequently, in preventing complications associated with intradialytic volume management.

Since predialysis BM is also used as an input for volume assessment by bioimpedance analysis, an inaccurate predialysis BM determination could lead to imprecise volume data as well. The error in the estimation of volume overload is, however, negligible: for the average dialysis patient (175 cm) with typical resistances R0 = 536 and Ri = 1740 [12] the difference in extracellular volume excess using either BM-reported or BM-undressed body mass inputs [13, 14], the difference in volume excess is only 20 mL. Nonetheless, the error in estimated fat mass is substantial and proportional to the error in body mass estimation.

The potential impact of seasonal variations in clothing mass must be emphasized [15]. Patients may arrive with heavier attire during colder seasons compared with warmer months. If this variation goes unaccounted for, seasonality may influence the accuracy of predialysis BM determination. Future research should address this seasonal aspect to provide a more comprehensive understanding of how clothing mass fluctuates throughout the year, contributing to a more refined and precise approach in HD settings.

It is important to acknowledge the limitations of our study. A *post hoc* power analysis indicated a high power of 1.0, suggesting the detection of the observed effect was likely. However, the study was conducted at a single center, was not representative of immobilized patients and was of moderate sample size, which may affect the generalizability of the findings. Our study's cross-sectional design prevents a detailed exploration of longitudinal clothing mass variability. We emphasize that longitudinal measurements, considering factors like seasons and changing clothing habits and calculating their exact impact on ultrafiltration goals, would offer a more nuanced understanding. The small sample size and the relevance of these findings was questioned during the review process, which is why we additionally conducted the online survey. Our results clearly showed that the weighing process was perceived to be important for dialysis tolerability among respondents. Almost 40% of participants reported frequent unintentional errors during weighing due to variations in clothing or accessories, corroborating that weighing was not a singular problem at our particular center.

In conclusion, our study highlights the potential impact of clothing mass on the accuracy of predialysis BM determination and subsequent implications for ultrafiltration management. The use of a standardized template for clothing mass demonstrated a clear advantage in reducing deviations and may contribute to more precise ultrafiltration goals. Further research is warranted to validate and extend our findings, ultimately improving the standardization of predialysis BM assessment in HD units with potential benefits to survival and morbidity.

## Data Availability

The data underlying this article will be shared upon reasonable request to the corresponding author.
